# Understanding Linkage Rules in Plant-Pollinator Networks by Using Hierarchical Models That Incorporate Pollinator Detectability and Plant Traits

**DOI:** 10.1371/journal.pone.0069200

**Published:** 2013-07-01

**Authors:** Ignasi Bartomeus

**Affiliations:** Swedish University of Agricultural Sciences, Department of Ecology, Uppsala, Sweden; University of Marburg, Germany

**Keywords:** Connectance, Linkage density, Nestedness, pollination web, specialization, hierarchical models, occupancy models

## Abstract

The analysis of mutualistic networks has become a central tool in answering theoretical and applied questions regarding our understanding of ecological processes. Significant gaps in knowledge do however need to be bridged in order to effectively and accurately be able to describe networks. Main concern are the incorporation of species level information, accounting for sampling limitations and understanding linkage rules. Here I propose a simple method to combine plant pollinator effort-limited sampling with information about plant community to gain understanding of what drives linkage rules, while accounting for possible undetected linkages. I use hierarchical models to estimate the probability of detection of each plant-pollinator interaction in 12 Mediterranean plant-pollinator networks. As it is possible to incorporate plant traits as co-variables in the models, this method has the potential to be used for predictive purposes, such as identifying undetected links among existing species, as well as potential interactions with new plant species. Results show that pollinator detectability is very skewed and usually low. Nevertheless, 84% of the models are enhanced by the inclusion of co-variables, with flower abundance and inflorescence type being the most commonly retained co-variables. The predicted networks increase network Connectance by 13%, but not Nestedness, which is known to be robust to sampling effects. However, 46% of the pollinator interactions in the studied networks comprised a single observation and hence could not be modeled. The hierarchical modeling approach suggested here is highly flexible and can be used on binary or frequency networks, accommodate different observers or include collection day weather variables as confounding factors. An R script is provided for a rapid adoption of this method.

## Introduction

Complex networks of biotic interactions such as predation, parasitism and pollination play an important role in shaping and maintaining biodiversity [1]. They can mediate important ecosystem functions, including those ecosystem services on which human well-being is dependent [2]. Our understanding of the structure and functioning of interactions has been accelerated by the use of network theory [1,3]. In particular, mutualistic interactions such as pollination, has received a lot of attention due to their functional importance. Mutualistic interaction networks are not randomly organized, but present some common properties. For example, the number of links per species tends to follow a truncated power law distribution [4] and the interaction web is usually nested, meaning that there is a core of generalist plant and pollinator species, and an asymmetric dependence between them [5,6]. As a consequence, plant-pollinator networks tend to be more robust to simulated extinctions than random networks [7].

Despite the clear benefits of using a network approach to investigate mutualistic interactions at the community level [8–13], limitations are evident [14–16]. First, the structure of plant-pollinator networks implies that the number of possible interactions increases exponentially with network size. Accurately detecting all interactions that are occurring in realistic large networks would require an enormous sampling effort, which is beyond the budget and time allocation of most projects [17]. In fact, the few studies that evaluate the sampling completeness of a plant-pollinator networks show that after four years of sampling, networks had not reached saturation [18]. Although many principal network metrics are robust to sampling bias [19,20], under-sampled communities may produce results that are difficult to interpret [15,21]. This is of less importance when comparisons among networks are relative, but under-sampling becomes more important when attempting to describe species roles within a network, such as specialization, or to predict ecological processes, such as susceptibility to extinction [7]. For example, rarity can be confounded with specialization and /or may drive observed patterns if overlooked [21,22]. Second, some researchers have claimed that network analysis is usually decoupled from ecological relevance [15], in that species are treated as nodes regardless of their life history characteristics. For example, despite the fact that flower morphology, abundance and rewards offered may influence attractiveness to pollinators [23,24] this information is usually not incorporated into network analysises. However, the use of species traits to inform the linkage rules of plants and pollinators is a developing field [25–29].

Interaction detectability (i.e. the probability to detect an interaction between a plant and its pollinator) can be enhanced by a number of methods. While most communities are sampled with a phytocentric approach (i.e. observing flowers and recording animal visits), including a zoocentric approach (i.e. sampling pollinators and measuring pollen loads [30]) can increase detectability and thus network Connectance (i.e. the number of links realized out of all possible links [31]). Similarly, using pollen stored in bee nests to infer visitation can increase the number of links for those species [22]. These approaches, are however time consuming and not feasible for most bee species, some of which are too rare to collect in sufficient numbers or nest in inaccessible substrates. In addition, other statistical methods proposed to account for undetected links [32,33] require independent data on both plant and pollinators, or data collected at the individual level to be implemented. This information is usually not available in published networks.

The probability of detecting an organism in time and space (i.e. detectability) is an important issue in other fields of research, like wildlife management [34]. Assessing the occupancy by a given species in a patch (i.e. presence/absence or frequency of this species in a predefined area) is complicated because imperfect detection of individuals or species can introduce substantial measurement error and obscure underlying ecological relationships if ignored. Recently, however, a broad class of hierarchical models has been developed [35,36]. Those methods offer a unified framework for analysis by formally recognizing that observations are generated by a combination of (1) a state process determining abundance or species occurrence at each site and (2) a detection process that yields observations conditional on the state process. The model for the state process describes abundance or occurrence at each site, but due to imperfect detection, these quantities cannot be observed directly and are regarded as latent variables. Moreover, parameters governing occurrence and detection processes can be modeled as functions of covariates [37]. Because these models have a straightforward interpretation paralleling mechanisms under which the data arose (i.e. detectability), they have recently gained popularity. While these tools have been designed to deal with species’ occurrence and abundance, its translation to interaction occurrences or frequency is straightforward. Here, we treat plant species as sites that the pollinators occupy or do not occupy, in a given sampling event. The two main assumptions of the models are: (1) the interaction or interaction frequency of a given pollinator species with each plant species remains constant during a sampling season and (2) repeated sampling periods at each plant are independent. Plant-pollinator networks fulfill both of these assumptions as they are usually treated as static snapshots along short time periods (i.e. season), where several independent sampling events are performed on each plant species. These models are advantageous in that instead of pooling interactions across sampling events on the same plant species, they account for multiple sampling events to estimate the probability of detecting a particular pollinator visiting each plant species. Further, the use of these estimated variables (i.e. detectability) enable estimation of the real number of interactions received by each plant.

This approach addresses significant knowledge gaps with respect to the structure of plant-animal interaction networks, and presents two major advantages over existing methods. First, the method allows for modeling the detection error of each species. Second, it enables the inclusion of covariates relating to collection conditions (e.g. site and weather information), and plant information (e.g. traits and abundance). Both plant abundance within the community and plant floral traits could determine attractiveness to different pollinators [21], but their relative contribution is not clear. The inclusion of these variables enhances the interpretation of model results and connects networks with relevant ecological information. These models are flexible in that they can be used on both presence-absence or abundance weighted data [35] and can use data obtained by standard collection methods.

Here, I use 12 published networks collected in Mediterranean shrub-lands to investigate the relative importance of covariates such as plant abundance and functional traits, to explain the plant-pollinator linkage rules, while accounting for pollinator species detectability. I then use the model outputs to predict the number of links between plants and pollinators and how this may modify network properties. Finally, I predict the response of such networks to potential changes in community composition, such as the invasion of an exotic plant species or simulated extinction patterns.

I ask three questions:

1. Can plant traits explain plant-pollinator interaction patterns (i.e. linkage rules)?
2. Is network structure robust to undetected links?
3. Can we predict pollinator visitation to new plant species (e.g. invasive species)?

## Material and Methods

• Networks used in this study:

I used 12 plant-pollinator networks sampled within Mediterranean scrublands in 2005 [38]. Each site was visited several times in consecutive weeks alternating visits in the morning and afternoon. Six coastal sites were sampled six times in March–April and the remaining six sites were sampled four times in May. Plant composition within sites did not change during this time frame. I randomly selected equal areas of target plants to observe each plant species for a total of six minutes per each round where visitation frequency of each pollinator species was recorded. All plants were sampled equally regardless of abundance. Voucher specimens of insect visitors were collected with a hand net for identification purposes after visitor observations were conducted. Plant flower abundance in the community was recorded in four 50 m transects. The mean flower abundance for each species at site level was used as covariates of the models in analyses. Flower units were defined as a single flower or an umbel, spike or capitulum on multi-flowered stems. Flower traits were gathered from the literature following Bosch et al. classification [19]. These included Inflorescence type (solitary, in raceme or composite flowers); Morphology (actinomorphic and zygomorphic); Shape (disc-bowl shaped corolla, restrictive tubular corolla, very restrictive papilionaceous corolla); and Colour (yellow, white, purple, green and blue). Data and traits are available at http://figshare.com/articles/Plant_Pollinator_Network_Data/154863 . Details on sampling protocol can be found in Bartomeus et al. [38].

In order to assess whether the identification of plant-pollinator interactions are dependent on sampling intensity, I calculated species accumulation curves with a Chao 2 estimator, both for the number of pollinator species sampled and the number of interactions sampled [18]. The difference between observed and estimated species richness or number of interactions was calculated as a percentage following Chacoff et al. [18].

• Statistical analysis:

### Hierarchical modeling approach

All analysis was performed at the pollinator species level. For each pollinator species in each network, I constructed an occupancy matrix comprising the recorded number of visits to each plant species in each sampling round. This is a common sampling design for obtaining interaction frequency information, where the researcher repeatedly visits a sample of all flowering plant species a number of times and record the number of unique individuals observed at each plant species for each time. I used the above-described matrices to fit hierarchical models using the function *pcount* in the package “Unmarked” in R [35]. This function uses repeated counts as detailed in Royle [39]. Our frequency data was fitted with a Poisson distribution, but “Unmarked” also supports negative binomial family distributions [35]. Abundance is modeled through a log link to enforce its positivity constraint and inference is based on the integrated likelihood wherein the latent state variable (i.e. interaction abundance) is marginalized out of the conditional likelihood [35]. The model output is composed by an estimate that defines the detection probability, and an estimate for each co-variable included in the model. These estimates are interpreted as in linear models and can be used to assess the effect sizes of each co-variable. Hierarchical models are highly flexible, and can also be used with binary networks (see function *occu* in Unmarked package). A complete explanation of the hierarchical models and the package used can be found in Fiske and Chandler [35], including tests of its performance under simulated scenarios.

Across the 12 networks, I identified 347 pollinator species. Although some of these pollinator species overlapped across networks, analyses were conducted independently for each network. Of these, 46% were singletons whereby a single individual of a pollinator species visited a single plant species. These were not analyzed further with hierarchical models. Overall, 186 models were analyzed, however, when re-constructing the estimated networks, the singletons were re-introduced and hence are included in all network metric calculations.

### Explaining linkage rules with plant traits

I explored a set of models including all co-variable combinations. In order to test if plant family can be used as a single predictor that captures floral trait differences, I also conducted alternative and simpler models by substituting all plant traits with the plant family information. Then, the model with the lowest AIC was selected [40]. These models estimate the pollinator species detectability, the probability that the pollinator will visit each plant species (i.e. probability of interactions, or occupancy in wildlife management literature) and how this probability is influenced by the co-variables retained in the model. For pollinators collected just once, the models cannot be calculated. An example R code of the analysis is provided in the Text S1.

### Estimating missing links and network parameters

To estimate the non-detected links, the output of the models was used to predict visitation to the plant community. Hence, the estimates of the retained co-variables in each model were used to determine the estimated interaction frequency. Network parameters of each observed and estimated network were calculated using the bipartite R-package [20]. These include three commonly used qualitative indexes (i.e. based on binary networks): Connectance, Linkage density and Nestedness; and two quantitative indexes (i.e. based on frequency networks); Specialization at the network level (H2’) and specialization at the pollinator species level (d') [41]. These indexes were chosen to represent a variety of commonly used indexes. Finally, I calculated robustness to higher or lower level extinctions[41]. I performed paired t-tests to assess the differences between observed and estimated networks.

### Predicting visitation to exotic plants

Six of the 12 networks have been invaded by an exotic plant (three by *Carpobrotus* *aff. acinaciformis* and three by *Opuntia* *stricta* ). I used this information to predict the likelihood that pollinator species in the non-invaded networks would visit a hypothetical exotic plant by predicting the visitation to a new plant with the exotic characteristics. I then compared to what extent the predicted links matched the observed links in the invaded network for the set of pollinator species present in both networks.

## Results

**Figure 1:**
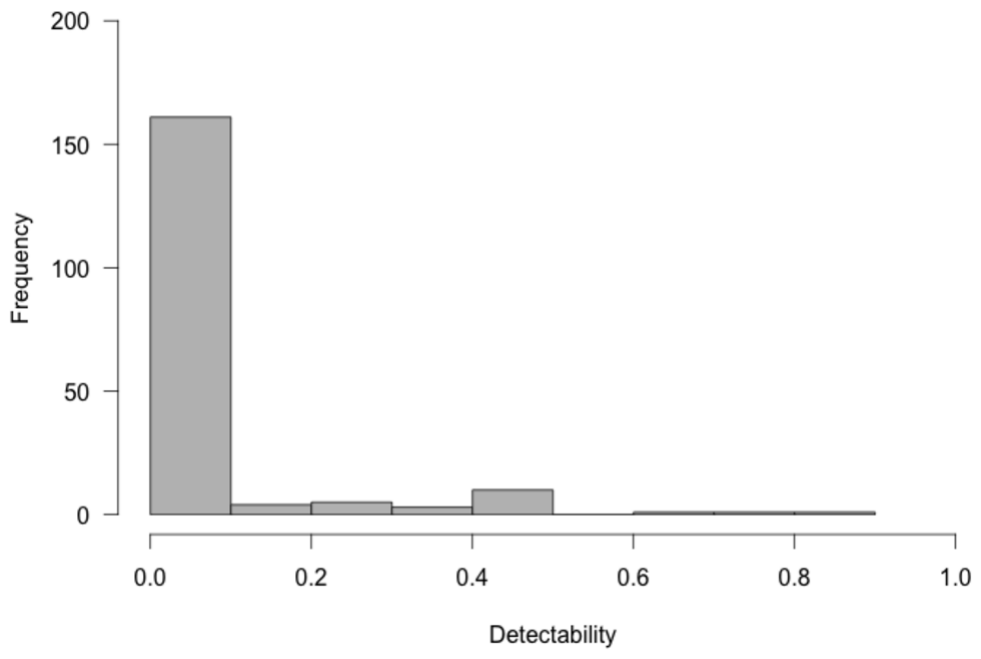
Histogram of the Detectability of each pollinator modeled. Despite some pollinator species show very high detectability, most show values below 10%.

The observed network mean size was 9.4 plant x 29.6 pollinator species, which is typical of published network sizes (i.e. 10 x 26; reviewed in 21). For pollinators, accumulations curves were close to saturation and I detected a mean of 72% of the estimated pollinator species in our networks but only 59% of the estimated interactions were detected. Hence, our networks are good candidates on which to test hierarchical models that account for non-detected links. Note that this under-sampling of interactions is common even in highly sampled plant-pollinator networks [18]. The detectability of analyzed pollinator interactions was very skewed (Figure 1), and few pollinators had detectabilities at over 10%. The proportion of plants visited (i.e. occupancy), detectability and visitation frequency recorded were not correlated (Pearson correlation range from 0.03 to 0.11; p-value > 0.05).

### Explaining linkage rules with plant traits

**Figure 2:**
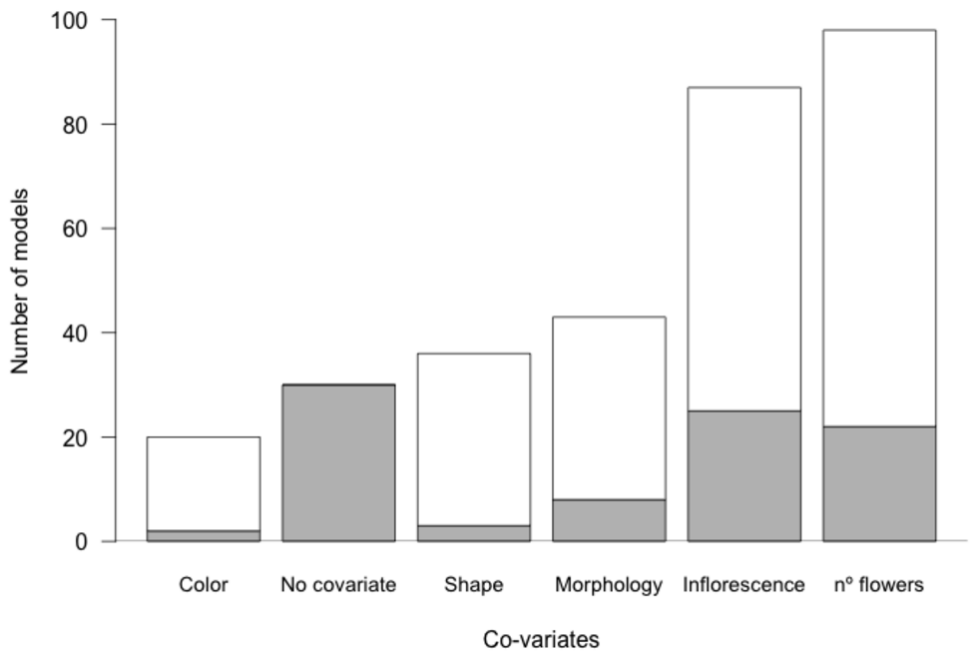
Frequency distribution of the number of models including five different potential co-variables of pollinator detectability (plus no covariate). In grey the proportion of models retaining only one co-variable. Note that most models retained more than one co-variable (186 models; median of 2 variables retained; 30 models retained no co-variables).

The 186 individual pollinator-network models retained a median of 2 co-variables (range 0-4; no model retained all possible 5 co-variables). Only 16% of the models did not retain any co-variables. Most models included the number of flowers and inflorescence type as important predictors for visitation, while color was rarely retained (Figure 2). Surprisingly, none of the models where plant family was included were selected as the best-fit model, indicating that in these networks, family is a poor substitute for plant traits.

### Estimating missing links and network parameters

**Figure 3:**
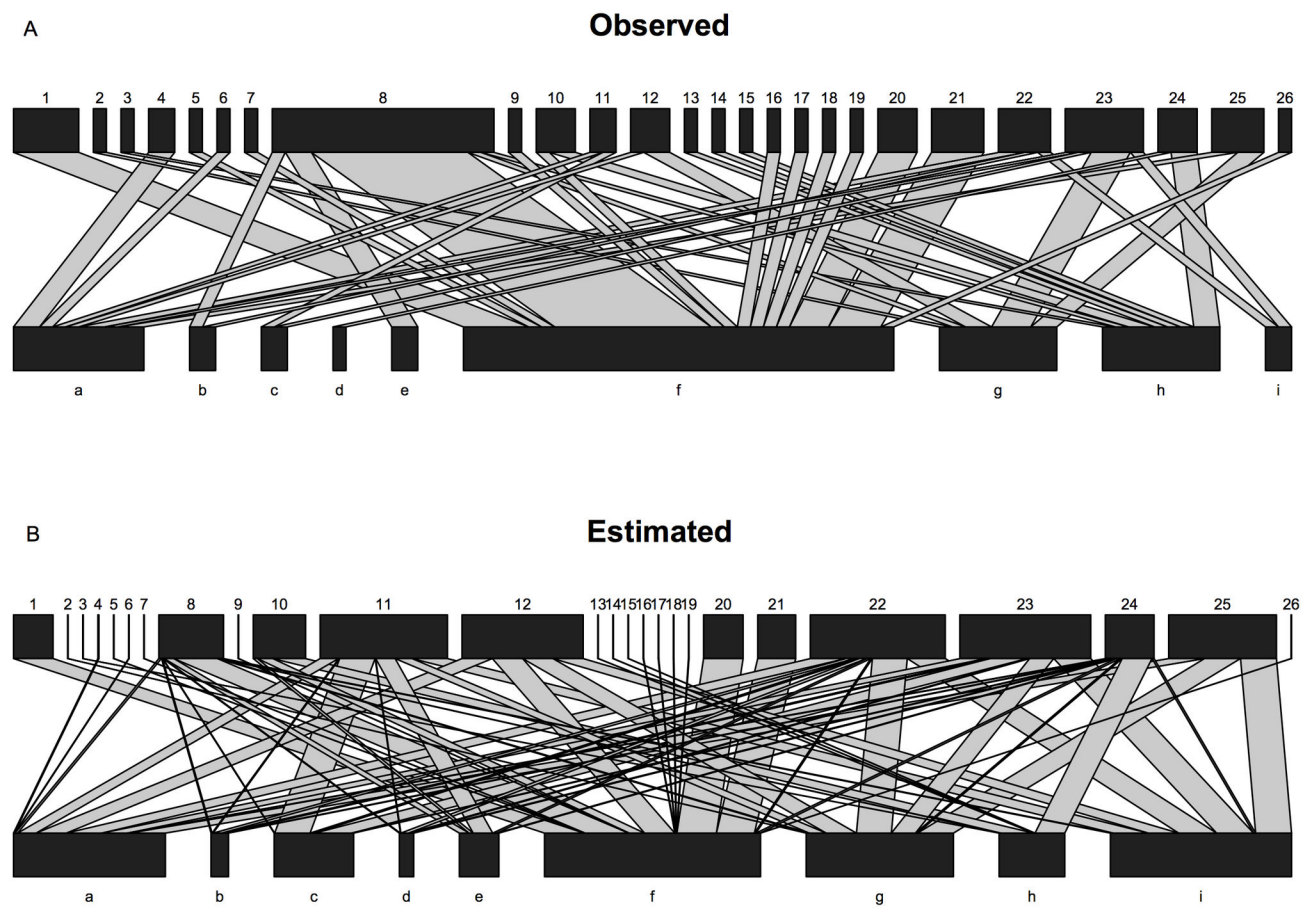
Observed and estimated visualization for one network (network MED2 in Bartomeus et al. 2008). Pollinators (numbers) are represented in the upper level, and plants (letters) in the lower level. Box size is proportional to the total number of visits recorded, and the link size to the frequency of this particular link. A) Observed data. B) Links estimated with the hierarchical models, which incorporate pollinator detectabilities.

**Figure 4:**
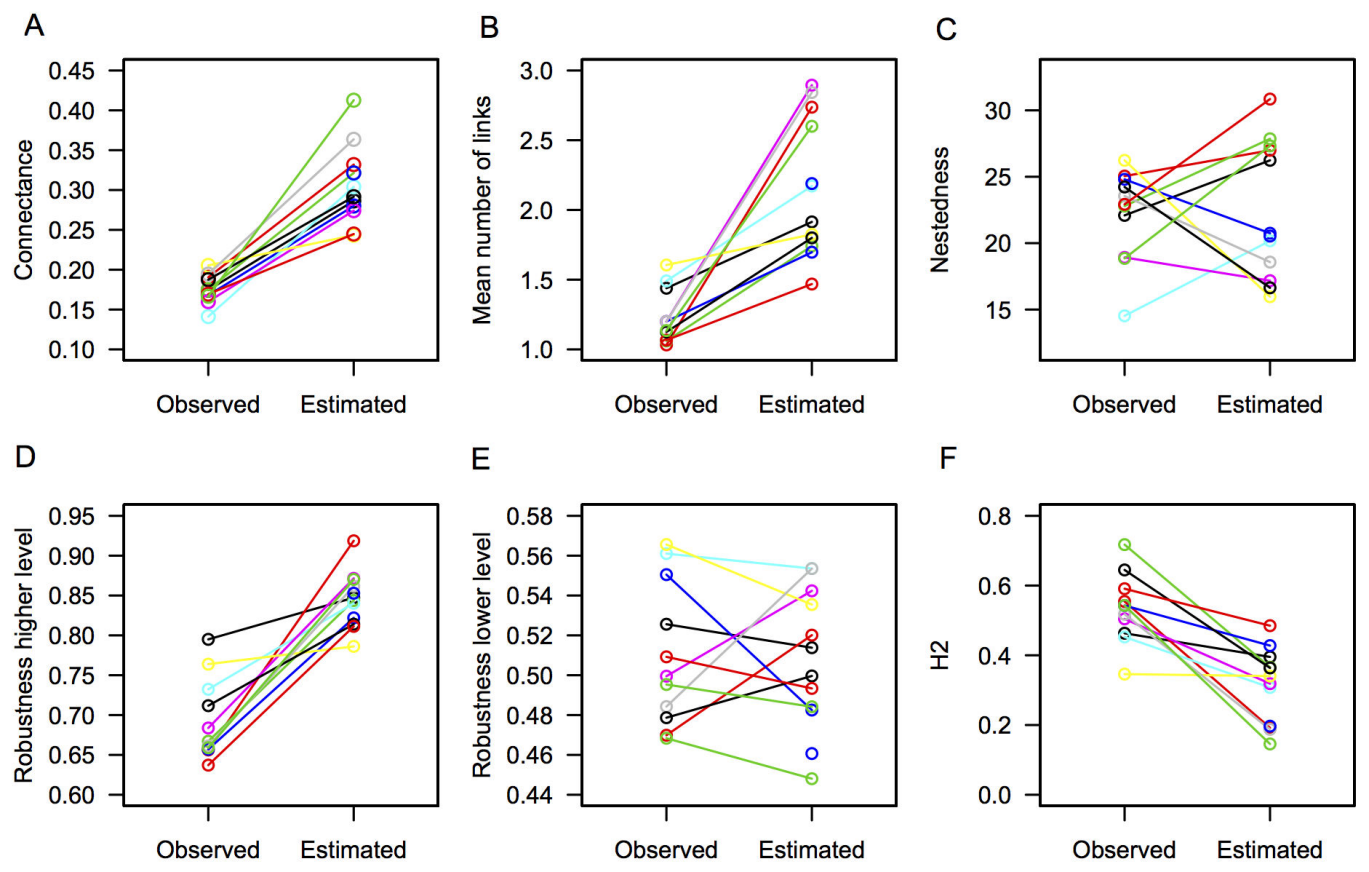
Network parameters of the observed networks, and its paired estimated network. Different colors are used for visualization purposes. A) Connectance, B) Linkage density, C) Nestedness, D) Robustness to pollinators extinction, E) Robustness to Plant extinctions and F) Specialization H2 index.

When I used model output to estimate the visitation frequency of each plant species and constructed the estimated networks (Figure 3), I found that estimated networks had a higher Connectance (p < 0.001) and Linkage density (p < 0.001) than observed networks. Nestedness however was not significantly different (p = 0.61). The specialization indexes (H2 and d') were lower in estimated networks (p < 0.001, p = 0.001). Robustness to plant extinctions was not different (p = 0.32), but robustness to pollinator extinctions increased significantly when comparing estimated and observed networks (p < 0.001; Figure 4).

### Predicting visitation to exotic plants

Of the pollinators present in non-invaded networks, 35% were predicted to visit the invader according to the exotic plant traits and abundance. For the pollinator species present in both non-invaded and invaded networks, 64% of the predicted interactions with the exotic plant were correct.

## Discussion

The probability of detecting an interaction in a given sampling event is lower than 10% for most interactions. This is a common pattern in plant-pollinator network studies [18]. This low detection probability has resulted in the pooling of observed interactions across several repeated sampling events. In this study I show that quantifying and incorporating this detectability into models to estimate interactions enables a greater understanding of the observed patterns. First, I show that plant traits and flower abundance can help explain linkage rules in most cases. For example, most pollinators respond to flower abundance independently of other floral traits (22/186 models), or in combination with inflorescence type (50/186; Figure 2). Moreover, when using this knowledge to estimate missing links, I confirm that some network properties, like Nestedness, are robust to sampling intensity [19] but also that specialization levels and network robustness may be more sensitive [16].

Interactions between plants and pollinators depend on a variety of factors, such as pollinator species preference [42] and how the community context (e.g. abundance, density and spatial distribution of flowers) modulates these preferences [43,44]. Floral traits are known to determine pollinator preferences [23]. Flower abundance, for example may be particularly relevant for social species that recruit workers [45]. In these Mediterranean networks, which include few strictly specialist species, flower abundance is the best predictor for explaining most pollinator interactions. The sampling methods used to sample each plant species were standardized [46], i.e. each species was observed for the same amount of time. Hence, the observed higher attractiveness for abundant flowers is not an artifact of greater sampling effort to those flowers, but a reflection of pollinator preference.

Other floral traits were also relevant in shaping the studied networks, namely inflorescence type and morphology. Both inflorescence type and morphology need different approaches and handling strategies by pollinators [47]. Colour was only relevant in a few models (Figure 2), probably due to the fact that the colors as viewed by insects are different from those captured in this analysis [48]. Surprisingly, plant family is a very poor predictor of visitation. This may not be the case in a community of many specialist species, but in our generalized community, plant traits are better predictors than plant family. Despite the fact that some traits are expected to be phylogenetically correlated [49], the results of this study do not support the use of plant family as an indicator of pollinator visitation. Despite the high turnover among the pollinators observed across networks, some pollinator species are represented in several networks, enabling a greater understanding of the generality of their associations with plant traits. Social pollinators (*Apis mellifera* and *Bombus* *terrestris* ) were consistently influenced by flower abundance (in 5/6 networks for *A. mellifera* and 9/12 for *B* *. terrestris* ). However, other species, like *Andrena* sp10, were more variable in their behavior (in 2/7 networks were influenced by traits, in 2/7 by floral abundance and in 3/7 no co-variables were selected). This variability may be explained by the context and is consistent with other studies showing species with inconsistent floral preferences among communities [42].

Second, I show that incorporating detectability to estimate missing links, resulted in an increase in Connectance and Linkage density. For example Connectance increased by 13% on average. Similarly, the linkage density estimated (mean = 2.27) is more similar to the expected value in other networks of similar size (i.e. 2.1; reviewed in 21) than the observed values. Nestedness, however did not change. Nestedness has previously been found to be one of the most robust metrics to characterize networks, and is not highly sensitive to sampling [19]. The degree of specialization decreased as a result of adding these new links. Results are consistent with the trends reported by Bosch et al. [23] when incorporating a zoocentric approach (see also 16).

A common approach to understand robustness to disturbance is to remove species randomly and see how many extinctions this produce. This metric is highly sensible to sampling completeness, as well as “rewiring strategies” (i.e. ability of pollinators to visit another plant when its preferred plant is not present [50]). Adding the estimated links can be seen both as increasing network completeness or accounting for possible rewiring of links in case the preferred plant goes extinct. In any case, we show that estimated networks are significantly more robust to pollinator extinctions. Moreover, estimated robustness values are very high (mean = 0.91; values range from 0 to 1), indicating that nearly all pollinator species need to be removed to identify effects at the plant level. However, robustness to plant extinctions do not change because these networks have a great amount of singletons (46% overall), which are not included in the detectability modeling, but are included into the estimated network. All singletons are consequently counted as being extinct when their unique partner is removed. This has a large influence on robustness patterns to plant extinctions, which has been a common problem in most studies to date [51].

Gibson et al [52] recently suggested that plant traits can be used to predict exotic species integration into an existing plant pollinator network (see also 53), such as when new plant invaders enter the community. My analysis supports this finding. I show that the estimated interactions of non-invaded communities to exotic plants are accurately predicted in 64% of the modeled interactions. This accuracy is reasonable high, considering that I am comparing the predicted responses to observations in a paired invaded community. Further, the natural variability in plant pollinator interactions among sites is usually high and the observed invaded community is probably under-sampled.

There are limitations in the use of hierarchical models for this purpose. First, singletons are not possible to model. This reduces the species pool that can be analyzed, but do not affect the models for the analyzed species. However, by explicitly targeting these species, researchers are forced to take singletons into account when interpreting and discussing results. Second, as in all trait-based approaches, the selection of the traits used can affect the results. For example, the inclusion of flower color as seen by insects, or nectar quality was not available for this study, but could enhance model predictions. Finally, the assumptions of the model imply that the different rounds of sampling should be completed within a short time period, to avoid changes in community composition over time.

This method can be applied to networks collected over short time periods and where the pollinator species sampling completeness is high, but interaction sampling completeness is low. While relative comparisons of networks collected under the same protocols may not benefit from this method, there are several questions that can take full advantage of it. First, when study generalities with respect to pollinator plant preferences, including the detection probability in the models allows to remove part of the bias that undetected links can introduce when assessing which plant traits are driving the strength of the interactions. Second, when comparing networks with different sampling effort, estimated interactions can minimize the differences in detectability of interactions among networks. Third, when there is an interest in understanding the consequences of alterations to plant community composition (e.g. plant invasions, changes in plant evenness, etc.), the output of the models can be used for predictive purposes.

In conclusion, I show that hierarchical modeling may be used to incorporate detectability and other co-variables in analyses of plant-pollinator networks. Understanding linkage rules can increase our ability to operate under a predictive framework where linkage rules can be explored and undetected links can be estimated. Moreover, more complex questions can be addressed incorporating covariates to the estimation of detectability, such as morning/afternoon sampling, weather conditions or observer identity [37].

## Supporting Information

Text S1 R code to load the data and reproduce the hierarchical modeling on a set of networks.
(DOCX)
