# Supplementary material for: Understanding Linkage Rules in Plant-Pollinator Networks by Using Hierarchical Models That Incorporate Pollinator Detectability and Plant Traits
Source: PLoS One. 2013 Jul 1;8(7):e69200. doi: 10.1371/journal.pone.0069200 (PMC3698228; doi:10.1371/journal.pone.0069200)
Supplement: Text S1 — R code to load the data and reproduce the hierarchical modeling on a set of networks. (DOCX) [file pone.0069200.s001.docx]

**Text S1**: R code to load the data and reproduce the hierarchical modeling on a set of networks.

#This code implements the analysis presented in

#"Understanding linkage rules in plant-pollinator networks

#by using hierarchical models that incorporate pollinator

#detectability and plant traits" by Ignasi Bartomeus

##Load the R libraries and the data required##

library(reshape)

library(bipartite)

library(unmarked)

#Data can be downloaded from the following webpage:

#[*http://figshare.com/articles/Plant_Pollinator_Network_Data/154863*](http://figshare.com/articles/Plant_Pollinator_Network_Data/154863)

d1 <- read.table("http://files.figshare.com/479751/Data_ntw_all.txt", h = TRUE)

#see the structure of the data

str(d1)

#extract plant trait variables from the datsset

traits <- unique(d1[,c(1,2,6,7,8,9,10,17)])

##Create some objects that will be needed for the analysis

#create a list of all Sites

site_list <- unique(d1$Site)

#create empty lists and data frames to store the main reults

est_ntws <- list()

ntws <- list()

Params <- data.frame(Site = NA, pol= NA, occup= NA, detect= NA, covar= NA, visits= NA, predCA = NA, predOP = NA, d_pol_raw = NA, d_pol_est = NA)

bipart <- data.frame(Site = NA, ntw = NA, pol = NA, plant= NA , conn = NA, links = NA, N = NA, NODF = NA, R_plants = NA, R_pol = NA, H2 = NA)

#create two hypothetical invasive species based on the characteristics of the real invasive species

#using SEL2OP Opuntia traits

traitsTemp <- subset(traits, Site == site_list[12])

new_spOP <- traitsTemp[which(traitsTemp$Plant_sp == "Opuntia_stricta"),]

#using MED2 Carpobrotus traits

traitsTemp <- subset(traits, Site == site_list[6])

new_spCA <- traitsTemp [which(traitsTemp $Plant_sp == "Carpobrotus_aff.acinaciformis"),]

##Creat a loop through all sites, and within each site, a second loop through all pollinators

#loop through the 12 sites

for (s in 1:12){

#select site data

d2 <- subset(d1, Site == site_list[s])

#construct the network

nw <- cast(d2, Plant_sp ~ Insect_sp, value = "Freq", fun = sum)

rownames(nw) <- nw[,1]

nw <- nw[-1]

#refactor the data

d2$Round <- as.factor(d2$Round)

d2$Plant_sp <- factor(d2$Plant_sp)

#make a vector with all pollinators

pol_list <- levels(factor(d2$Insect_sp))

#create an empty matrix to store the estimated network and the parameters:

estimated_Ntw <- matrix(ncol = length(pol_list), nrow = length(unique(d2$Plant_sp)), dimnames = dimnames(nw))

Pol_parameters <- data.frame(pol = pol_list, occup = c(1:length(pol_list)), detect = c(1:length(pol_list)), covar = c(1:length(pol_list)), visits = c(1:length(pol_list)), predCA = c(1:length(pol_list)), predOP = c(1:length(pol_list)))

#loop thought pollinators

for (i in 1:length(pol_list)){

#select each species

d1p1 <- subset(d2, Insect_sp == pol_list[i])

#Create the occupancy matrix (visits to each plant per round)

y <- cast(d1p1, Plant_sp ~ Round, value = "Freq", fun = sum, add.missing= TRUE)

y <- as.matrix(y[1:length(y[,1]),])

#Exclude Singletones from the analysis

if(sum(y) < 2){

#add NA in the Output data frame, as those are not analyzed

Pol_parameters[i,2] <- NA

Pol_parameters[i,3] <- NA

Pol_parameters[i,4] <- NA

Pol_parameters[i,5] <- sum(y)

Pol_parameters[i,6] <- NA

Pol_parameters[i,7] <- NA

estimated_Ntw[,i] <- nw[,i]

}

else {

#prepare the data for modeling

pl <- rownames(y)

traits2 <- subset(traits, Site == site_list[s])

covs <- traits2[which(traits2$Plant_sp %in% pl),]

#Format for unmarked and summarize data

umf <- unmarkedFramePCount(y=y, siteCovs= covs)

summary(umf)

# Fit the models

# Detection covariates follow first tilde, then come abundance covariates

#First all possible family and abundance models

lam <- pcount(~1 ~ 1, data=umf)

lam_Family <- pcount(~1 ~ P_Family, data=umf,se = FALSE)

lam_meanflw <- pcount(~1 ~ mean_flw, data=umf,se = FALSE)

lam_Family_meanflw <- pcount(~1 ~ P_Family + mean_flw, data=umf, se = FALSE)

#Then all possible traits models

#one by one

lam_Morphology <- pcount(~1 ~ P_Morphology, data=umf,se = FALSE)

lam_Inflorescence <- pcount(~1 ~ P_Inflorescence, data=umf,se = FALSE)

lam_Shape <- pcount(~1 ~ P_Shape, data=umf,se = FALSE)

lam_Color <- pcount(~1 ~ P_Color, data=umf,se = FALSE)

#all traits

lam_Morphology_Inflorescence_Shape_Color <- pcount(~1 ~ P_Morphology + P_Inflorescence + P_Shape + P_Color, data=umf,se = FALSE)

#Combinations of 3 Traits

lam_Morphology_Inflorescence_Shape <- pcount(~1 ~ P_Morphology + P_Inflorescence + P_Shape, data=umf,se = FALSE)

lam_Morphology_Inflorescence_Color <- pcount(~1 ~ P_Morphology + P_Inflorescence + P_Color, data=umf,se = FALSE)

lam_Morphology_Shape_Color <- pcount(~1 ~ P_Morphology + P_Shape + P_Color, data=umf,se = FALSE)

lam_Inflorescence_Shape_Color <- pcount(~1 ~ P_Inflorescence + P_Shape + P_Color, data=umf,se = FALSE)

#Combinations of 2 Traits

lam_Morphology_Inflorescence <- pcount(~1 ~ P_Morphology + P_Inflorescence, data=umf,se = FALSE)

lam_Shape_Color <- pcount(~1 ~ P_Shape + P_Color, data=umf,se = FALSE)

lam_Morphology_Color <- pcount(~1 ~ P_Morphology + P_Color, data=umf,se = FALSE)

lam_Inflorescence_Color <- pcount(~1 ~ P_Inflorescence + P_Color, data=umf,se = FALSE)

lam_Morphology_Shape <- pcount(~1 ~ P_Morphology + P_Shape, data=umf,se = FALSE)

lam_Inflorescence_Shape <- pcount(~1 ~ P_Inflorescence + P_Shape, data=umf,se = FALSE)

#Combination of each traits and abundance

lam_Morphology_meanflw <- pcount(~1 ~ P_Morphology + mean_flw, data=umf, se = FALSE)

lam_Inflorescence_meanflw <- pcount(~1 ~ P_Inflorescence + mean_flw, data=umf,se = FALSE)

lam_Shape_meanflw <- pcount(~1 ~ P_Shape + mean_flw, data=umf, se = FALSE)

lam_Color_meanflw <- pcount(~1 ~ P_Color + mean_flw, data=umf, se = FALSE)

#Combination of 2 Traits and abundance

lam_Morphology_Inflorescence_meanflw <- pcount(~1 ~ P_Morphology + P_Inflorescence + mean_flw, data=umf,se = FALSE)

lam_Shape_Color_meanflw <- pcount(~1 ~ P_Shape + P_Color + mean_flw, data=umf,se = FALSE)

lam_Morphology_Color_meanflw <- pcount(~1 ~ P_Morphology + P_Color + mean_flw, data=umf,se = FALSE)

lam_Inflorescence_Color_meanflw <- pcount(~1 ~ P_Inflorescence + P_Color + mean_flw, data=umf,se = FALSE)

lam_Morphology_Shape_meanflw <- pcount(~1 ~ P_Morphology + P_Shape + mean_flw, data=umf,se = FALSE)

lam_Inflorescence_Shape_meanflw <- pcount(~1 ~ P_Inflorescence + P_Shape + mean_flw, data=umf,se = FALSE)

#Combination of 3 Traits and abundance

lam_Morphology_Inflorescence_Shape_meanflw <- pcount(~1 ~ P_Morphology + P_Inflorescence + P_Shape + mean_flw, data=umf,se = FALSE)

lam_Morphology_Inflorescence_Color_meanflw <- pcount(~1 ~ P_Morphology + P_Inflorescence + P_Color + mean_flw, data=umf,se = FALSE)

lam_Morphology_Shape_Color_meanflw <- pcount(~1 ~ P_Morphology + P_Shape + P_Color + mean_flw, data=umf,se = FALSE)

lam_Inflorescence_Shape_Color_meanflw <- pcount(~1 ~ P_Inflorescence + P_Shape + P_Color + mean_flw, data=umf,se = FALSE)

#All traits and abundance

lam_Morphology_Inflorescence_Shape_Color_meanflw <- pcount(~1 ~ P_Morphology + P_Inflorescence + P_Shape + P_Color + mean_flw, data=umf,se = FALSE)

#create a list of all candidate models

ms<- fitList(lam, lam_Family, lam_meanflw, lam_Family_meanflw, lam_Morphology, lam_Inflorescence, lam_Shape, lam_Color, lam_Morphology_Inflorescence_Shape_Color, lam_Morphology_Inflorescence_Shape, lam_Morphology_Inflorescence_Color, lam_Morphology_Shape_Color, lam_Inflorescence_Shape_Color, lam_Morphology_Inflorescence, lam_Shape_Color, lam_Morphology_Color, lam_Inflorescence_Color, lam_Morphology_Shape, lam_Inflorescence_Shape, lam_Morphology_meanflw, lam_Inflorescence_meanflw, lam_Shape_meanflw, lam_Color_meanflw, lam_Morphology_Inflorescence_meanflw, lam_Shape_Color_meanflw, lam_Morphology_Color_meanflw, lam_Inflorescence_Color_meanflw, lam_Morphology_Shape_meanflw, lam_Inflorescence_Shape_meanflw, lam_Morphology_Inflorescence_Shape_meanflw, lam_Morphology_Inflorescence_Color_meanflw, lam_Morphology_Shape_Color_meanflw, lam_Inflorescence_Shape_Color_meanflw, lam_Morphology_Inflorescence_Shape_Color_meanflw)

# Rank them by AIC

ms1 <- modSel(ms)

#Select best model

model <- ms1@Full$model[1]

#And extract estimates

m <- ms@fits[model][[1]]

beta1 <- coef(m)

# Note, estimates of detection coefficients are on the logit-scale

#store results on the Output data frame

Pol_parameters[i,2] <- plogis(beta1)["lam(Int)"]

Pol_parameters[i,3] <- plogis(beta1)["p(Int)"]

Pol_parameters[i,4] <- ms1@Full$model[1]

Pol_parameters[i,5] <- sum(y)

#calculate estimated visitation frequency and store it in the extimated network

estimated_Ntw[,i] <- round(predict(m, type="state", newdata=covs)$Predicted)

#predict visitation to the new species

Pol_parameters[i,6] <- round(predict(m, type="state", newdata=new_spCA)$Predicted)

Pol_parameters[i,7] <- round(predict(m, type="state", newdata=new_spOP)$Predicted)

}

}

##store results at the community level

#get the complete raw network

nw2 <- as.matrix(nw)

dimnames(nw2) = dimnames(nw)

#store it in the list ntws

ntws[[s]] <- nw2

#fill site name

Pol_parameters$Site <- rep(site_list[s], length(Pol_parameters[,1]))

sp <- specieslevel(nw2)

#Add singletones back

for(a in 1:length(estimated_Ntw[1,])){

if (sum(estimated_Ntw[,a]) < 1){

estimated_Ntw[,a] <- nw2[,a]

}

}

#store network metrics using bipartite package fucntions specieslevel

spe <- specieslevel(estimated_Ntw)

#number of pollinators raw community

d_pol_raw <- as.data.frame(sp$`higher trophic level`$d, optional = TRUE)

colnames(d_pol_raw) <- c("d_pol_raw")

Pol_parameters$d_pol_raw <- d_pol_raw$d_pol_raw

#number of pollinators estimated community

d_pol_est <- as.data.frame(spe$`higher trophic level`$d, optional = TRUE)

colnames(d_pol_est) <- c("d_pol_est")

Pol_parameters$d_pol_est <- d_pol_est$d_pol_est

#store the full estimated community

est_ntws[[s]] <- estimated_Ntw

#Add the parameters to the Output

Params <- rbind(Params,Pol_parameters)

str(Pol_parameters)

#Calculate parameters using networklevel function on bipartite package

n <- networklevel(nw2)

ne <- networklevel(estimated_Ntw)

#store them

bipart_params_n <- data.frame(Site = rep(site_list[s], 1), ntw = rep("original", 1), pol = n$`number of higher trophic species`, plant= n$`number of lower trophic species`, conn = n$connectance, links = n$`links per species`, N = n$nestedness, NODF = n$`weighted NODF`, R_plants = n$`robustness lower exterminated`, R_pol = n$`robustness higher exterminated`, H2 = n$H2)

bipart_params_ne <- data.frame(Site = rep(site_list[s], 1), ntw = rep("estimated", 1), pol = ne$`number of higher trophic species`, plant= ne$`number of lower trophic species`, conn = ne$connectance, links = ne$`links per species`, N = ne$nestedness, NODF = ne$`weighted NODF`, R_plants = ne$`robustness lower exterminated`, R_pol = ne$`robustness higher exterminated`, H2 = ne$H2)

bipart <- rbind(bipart,bipart_params_n, bipart_params_ne)

#As is a slow loop, we can print the network number (s) to keep track

print(s)

}

#clean the Output

Params <- Params[-1,]

bipart <- bipart[-1,]

#See the Output:

est_ntws #list of estimated Networks; access to the first one by est_ntw[[1]]

ntws #idem for observed networks

Params #data frame of the parameters of all models

bipart #data frame of the network metrics for each network
